# Perceived remuneration fairness, career growth and turnover intentions among female engineers in a South African organisation

**DOI:** 10.3389/fsoc.2026.1917717

**Published:** 2026-10-14

**Authors:** MaryAnne Williams, Mokgata Alleen Matjie, Sonja Grobler

**Affiliations:** Department of Industrial and Organisational Psychology, School of Management Sciences, University of South Africa, Pretoria, South Africa

**Keywords:** perceived remuneration fairness, career growth, turnover intentions, female engineers, gender equity, engineering, South Africa

## Abstract

**Introduction:**

Gender inequalities in engineering remain an important organisational concern, particularly in relation to remuneration fairness, career growth and employees’ intentions to remain with or leave an organisation. Female engineers may evaluate their workplace experiences not only in terms of remuneration, but also in relation to opportunities for professional development, advancement, meaningful work and organisational support. This study explored how female engineers perceive remuneration fairness and career growth and how these experiences relate to their turnover intentions within a South African engineering organisation.

**Methods:**

The study adopted an interpretivist epistemological orientation and a qualitative phenomenological research design to explore participants’ lived experiences and interpretations of remuneration, career growth and intentions to remain with or leave the organisation. Data were collected through semi-structured individual interviews with nine female engineers and analysed using inductive thematic analysis.

**Results:**

Participants’ perceptions of remuneration fairness varied according to individual expectations, comparison points, workload, career aspirations and interactions with management. While some participants regarded their remuneration as fair or acceptable, others expressed concerns regarding remuneration and organisational processes influencing pay outcomes. These perceptions should not, however, be interpreted as evidence of the absence of objective gender-based remuneration disparities. Career growth emerged as an important consideration, with participants highlighting opportunities for advancement, access to meaningful and technically challenging work, professional development, recognition and workplace flexibility. Experiences relating to remuneration and career growth were also associated with participants’ reflections on their intentions to remain in or consider leaving the organisation. Organisationally mediated negotiation was perceived by some participants as influencing remuneration outcomes, although the findings do not support generalisations about women’s negotiation abilities or willingness to accept inequitable remuneration.

**Discussion and conclusion:**

The findings suggest that perceived remuneration fairness and career growth are important dimensions of female engineers’ workplace experiences and may shape how they evaluate their future within an organisation. Turnover intentions should therefore be understood within the broader organisational context of remuneration practices, career-development opportunities, recognition, meaningful work and workplace support. The study emphasises the need to distinguish subjective perceptions of remuneration fairness from objective pay equity, which requires comparative remuneration data and consideration of factors such as job level, responsibilities, experience, performance and benefits. Organisations seeking to retain female engineering talent should strengthen transparent remuneration practices, equitable career-development pathways and supportive organisational conditions that facilitate advancement and long-term career commitment.

## Introduction

The Science, Technology, Engineering, and Mathematics (STEM) sectors are male-dominated in most countries (Wang and Degol, 2017; United Nations Educational and Scientific and Cultural Organization (UNESCO), 2024), including South Africa (Engineering Council of South Africa (ECSA), 2024; Hammond et al., 2020; Sibisi et al., 2025; United Nations Educational and Scientific and Cultural Organization (UNESCO), 2024; Van Heuvelen and Quadlin, 2021). There is systematic gatekeeping of the sector for females who might be interested and qualify to work in the sector, of which the popular one is the deliberate unequal and unfair remuneration disparities (Hammond et al., 2020; Hunt, 2016; Ohunakin and Olugbade, 2022; Stofberg et al., 2022). These deliberate remuneration disparities are used to discourage females from remaining in the sector for long, thus exacerbating the high turnover of females from the sector (Lourens et al., 2025; Van Heuvelen and Quadlin, 2021), through socio-cultural norms, educational barriers, workplace inequalities, and limited participation at various levels (Sibisi et al., 2025), affecting their career growth and turnover intentions. This situation leads to an increased unemployment rate in South Africa, with the current statistics standing at 32.7% in May 2026 (Statistics South Africa (Stats SA), 2026). Theorist Adams (1965) proposed that employee motivation is driven by a desire for fairness, and when females specifically in the engineering sector experience unfairness and toxic masculinity tendencies, their motivation levels drop, and they intend to, and ultimately, leave the field (Fouad et al., 2017; González-Pérez et al., 2022; Hlotshana, 2024).

In South Africa, these disparities are compounded by broader socio-economic divides and historical inequities that continue to shape the labour market (Siwela and van der Bank, 2021). Within this context, female engineers represent a particularly under-researched group, despite the critical role they play in STEM workforce development (Lourens et al., 2025). Specific to the engineering sector, despite global and national efforts to promote gender equity in STEM fields, disparities in remuneration, career advancement, and retention of female engineers remain pervasive (Hammond et al., 2020). Research has consistently demonstrated that women in STEM earn significantly less than their male counterparts, with South African female engineers facing a 28% wage gap (Son Hing et al., 2023; Taylor, 2023), more specifically in the engineering sector (Van Heuvelen and Quadlin, 2021; Zajac et al., 2024). Beyond financial disparities, women in the engineering sector report limited access to leadership roles, biased salary negotiations, and fewer career growth opportunities, which exacerbates their perceptions of workplace inequity (Lourens et al., 2025; Van Heuvelen and Quadlin, 2021).

This study specifically focuses on females in the engineering sector because higher remuneration disparities were reported (Van Heuvelen and Quadlin, 2021; Zajac et al., 2024), with dire psychological consequences. Fairness in remuneration is a fundamental concept in workplace psychology and is closely tied to organisational justice theories (Taye et al., 2020). Employees assess fairness through procedural justice (the fairness of decision-making processes) and distributive justice (the fairness of actual outcomes) (Greenberg, 1987). Research has consistently demonstrated that when employees perceive unfair remuneration practices, they experience lower motivation, job dissatisfaction, and higher turnover intentions (Stofberg et al., 2022). Furthermore, the Equity Theory (Adams, 1963) suggests that employees determine fairness by comparing their input-to-output ratios with those of their peers (Mengstie, 2020). If they perceive inequity, they may reduce their effort, seek alternative employment, or become disengaged (Ohunakin and Olugbade, 2022). Research highlights that those perceived inequities in STEM compensation structures affect women and minority groups disproportionately, leading to higher attrition rates (Bae et al., 2022). Moreover, Blau (1964a, 1964b) noted that perceptions of fairness and support are fundamental predictors of retention outcomes, with unfair treatment linked to higher withdrawal cognitions and intentions to leave (Blau, 1964a, 1964b; Campbell et al., 2018). Despite these perceptions, remuneration disparities across genders persist. As a result, the current study aims to bring forth female engineers’ perceptions of and experiences of remuneration inequalities and how they influence their career growth and ultimately turnover intentions.

South Africa’s engineering sector is pivotal to the country’s socio-economic development, underpinning infrastructure development, energy systems, and industrial competitiveness. Despite its strategic importance, sustaining a skilled and diverse engineering workforce remains challenging amid persistent skills shortages, attrition within the engineering pipeline, and ongoing inequities in workplace experiences. Although there have been targeted efforts to bolster female participation in science, technology, engineering, and mathematics (STEM), women’s representation declines markedly at advanced stages of educational and professional careers. National data indicate that women constitute approximately 43–44% of STEM graduates, yet they represent only 23–29% of senior professional roles, reflecting the systemic attrition often described as the “leaky pipeline” (Bizcommunity, 2026).

Within the engineering profession specifically, gender imbalances are stark. According to the Engineering Council of South Africa (ECSA), women represent a small minority of registered professional engineers, with only about 14.2% of registered engineers identifying as female, despite incremental increases through concerted recruitment efforts (SME Tech Guru, 2024; Engineering Council of South Africa (ECSA), 2024). This underrepresentation reflects not only educational pipeline issues but also systemic challenges in recruitment, retention, and workplace culture, including limited access to mentorship and professional networks, as well as enduring perceptions of engineering as a male-dominated domain (South African Business Matters, 2025). According to Sibisi et al. (2025), women in STEM generally experience unequal pay, lack of mentorship, limited networking opportunities, and workplace discrimination.

A central dimension of workplace equity concerns remuneration fairness. Despite constitutional protections and legislative frameworks such as the Employment Equity Act aimed at reducing discrimination and promoting equitable practices, gender remuneration disparities persist across the South African labour market. Recent reports show that women, on average, earn significantly less than men, with gender remuneration gaps widening with age and seniority and profound implications for lifetime earnings and economic security (Cape Argus, 2025). Research also points to ongoing perceptions of inequity and opacity around remuneration practices (Hasyim and Bakri, 2024; Mabaso and Mdluli, 2025; Stofberg et al., 2022). Surveys of professional respondents highlight stark divergences in remuneration equity perceptions by gender, with a majority of men reporting confidence in equal remuneration policies while only a minority of women agree that salaries are equitable, and many women suspect that male colleagues receive higher compensation for comparable work (Engineering News, 2025). These discrepancies can erode trust in organisational processes, influence perceptions of organisational justice, and shape career aspirations and decisions.

In addition to remuneration, the broader labour market context in South Africa shapes career growth decisions. Persistent shortages of engineers create a competitive environment in which highly skilled professionals may have mobility options across sectors or even internationally. These dynamics underscore the importance of workforce integration and retention strategies to sustain economic competitiveness and mitigate brain drain, as noted in recent economic surveys (OECD, 2025). In such a market, perceived remuneration inequities may nudge professionals toward alternative opportunities that better align with their development goals.

Crucially, perceptions of remuneration fairness intersect with broader cultural and institutional factors influencing women’s participation in engineering. Gendered expectations related to caregiving responsibilities, limited representation in decision-making roles, and organisational cultures that privilege male norms can compound the effects of remuneration inequities, shaping career trajectories differently for female engineers compared with their male counterparts.

Given this context, exploring how female engineers experience and interpret fairness in remuneration and how these perceptions shape career decisions is both timely and critical. Such insights are essential for formulating interventions that promote equitable remuneration, strengthen organisational justice, and retain diverse talent in STEM professions, outcomes that are vital for fostering inclusive growth and innovation in South Africa.

Career growth was defined as the opportunity for individuals to gain career growth in an organization, such as undertaking challenging work tasks, taking on more job responsibilities, and receiving more remuneration (Graen et al., 1997). For female engineers, this implies promotions to a higher position because the line managers have faith in such an engineer and see potential. However, this is not always the case because line managers often become the stumbling block; careful planning of limited training and support, to frustrates female engineers, leading to low commitment and high turnover intentions (Abdalnour et al., 2023; Fouad et al., 2017; Nouri and Parker, 2013). Turnover intention refers to an employee’s likelihood of leaving their organisation due to dissatisfaction or perceived unfairness in the workplace (Taye et al., 2020). Studies show that turnover intentions increase significantly when employees feel undervalued or perceive inequity in pay and career advancement opportunities (Kamau and Omondi, 2020). In a construction company, it was found that active career growth opportunities lead to low turnover intention (Sasanti et al., 2024). Contrarily, New Zealand-based female engineers were found to be persistent in staying due to a sense of belonging, meaningfulness of work, psychological safety, and support from co-workers, and they remain career committed to the STEM sector (Djung et al., 2024). This implies that the potential for career growth is an essential motivating element for employees, including female engineers, reducing turnover intentions (Al Balushi et al., 2022). For female engineers, career growth serves as a perceived enhanced value that leads to commitment, which further reduces turnover intentions (Allen et al., 2003; Soenanta et al., 2020). Various theories may best explain how negative or positive perceptions and experiences influence career growth and turnover intentions.

The study is grounded in three complementary theoretical perspectives that explain how employees interpret equity or fairness in remuneration and how these perceptions influence career growth and turnover intentions. John Stacey Adams first articulated Equity Theory in the early 1960s to explain individuals’ perceptions of fairness in social exchange relationships at work. The core premise of Equity Theory is that employees evaluate the fairness of their remuneration by comparing their inputs (e.g., skills, effort, experience) and outcomes (e.g., remuneration, benefits, recognition) with those of a relevant referent other, such as a peer or colleague. When employees perceive an imbalance in these ratios, they experience psychological distress and are motivated to restore equity through behavioural or cognitive adjustments, including reducing effort, renegotiating rewards, or seeking alternative employment opportunities. The theory thus provides a strong lens for understanding why female engineers who perceive inequitable remuneration relative to peers may reassess their organisational commitment or consider career mobility to restore perceived fairness. Equity Theory remains one of the most influential frameworks in organisational research on fairness and has been supported across contexts (Adams, 1963, 1965; Cropanzano and Greenberg, 1997; Lee and Rhee, 2023).

Organisational Justice Theory (OJT) extends the concept of fairness by focusing on employees’ perceptions of fairness in organisational processes and outcomes, not just outcomes alone. Originating in work by Greenberg (1987), this theory distinguishes between three primary justice dimensions: distributive justice (fairness of outcome distribution such as remuneration), procedural justice (fairness of the processes used to decide those outcomes), and interactional justice (fairness in interpersonal treatment during implementation). In remuneration contexts, a lack of transparent benchmarking or processes that rely heavily on individual negotiation can undermine perceptions of procedural justice, leading employees to question the legitimacy and fairness of organisational decisions (Cruz et al., 2026; Lourens et al., 2025; Van Heuvelen and Quadlin, 2021; Zajac et al., 2024). Perceptions of organisational justice are linked to a range of work attitudes and behaviours, including job satisfaction, engagement, and turnover intentions (Colquitt et al., 2001; Pieters, 2016).

Social Exchange Theory (SET), as articulated by Blau (1964a, 1964b), emphasises that workplace relationships are built on reciprocal exchanges where each party provides something of value to the other. Employees who perceive fair treatment and equitable rewards from their organisation are likely to reciprocate with positive work behaviours, such as organisational commitment and intention to remain. Conversely, when employees feel unfairly treated or that the psychological contract has been breached, they may withhold effort, reduce commitment, or explore exit options. This reciprocal logic helps explain why perceived inequities in remuneration may reduce organisational attachment and increase turnover intentions among female engineers. Research grounded in social exchange principles has consistently shown that perceptions of fairness and support are fundamental predictors of retention outcomes, with unfair treatment linked to higher withdrawal cognitions and intentions to leave (Blau, 1964a, 1964b; Campbell et al., 2018).

Together, these theories provide a robust conceptual framework for the study. Equity Theory (ET) explains the cognitive evaluation of fairness based on comparisons. Organisational Justice Theory illuminates how procedural and interactional dimensions shape perceptions of fairness beyond raw outcomes (such as salary levels), and Social Exchange Theory provides the behavioural mechanism by which these fairness perceptions influence organisational commitment and career decisions. Collectively, they suggest that perceptions of remuneration equity are not just about remuneration levels but about how remuneration decisions are made and how they reflect the quality of the employee–organisation relationship. In the context of female engineers, this framework supports the conceptualisation that perceived inequities in remuneration may function as either positive or negative triggers of career growth and turnover intentions, mediated through fairness evaluations and reciprocal relational exchanges.

As per the thorough literature review, the study aimed to:

- Explore the female engineers’ perceptions and experiences of remuneration fairness and inequity, and how remuneration influences their retention and career progression.

## Methods

This study adopted a qualitative phenomenological research design (Tavakol and Sandars, 2025) to explore the lived experiences and perceptions of female engineers regarding remuneration fairness and career mobility within an engineering workplace in South Africa. A qualitative approach was considered appropriate, according to Tenny et al. (2022), because the study sought to understand how participants experienced, interpreted, and attached meaning to remuneration, organisational fairness, career development, and mobility within their workplace context. Rather than seeking to measure the prevalence or magnitude of gender-based remuneration disparities, the study focused on participants’ subjective experiences and perceptions of these issues (Tenny et al., 2022). The phenomenological orientation enabled the researchers to examine the meaning participants attributed to their workplace experiences and to identify commonalities across their accounts (Tavakol and Sandars, 2025). The study therefore treats participants’ accounts as situated experiences rather than as objective evidence of the organisation’s actual remuneration practices.

### Participants and setting

The engineering company employed approximately 250 staff members, including engineers; however, the specific number of female engineers was not disclosed. Given the qualitative and exploratory nature of the study, purposive sampling was employed to identify participants with relevant experience and knowledge of the phenomenon under investigation (Creswell and Poth, 2018; Palinkas et al., 2015). This sampling approach was appropriate for exploring the subjective experiences of female engineers, particularly their perceptions of remuneration fairness and workplace equity within a male-dominated STEM environment (Stratton, 2023).

Participants were recruited through an engineering professional association, the Engineering Council of South Africa (ECSA), with which practicing engineers are registered. Approval to carry out the study was granted by the gatekeeper, ECSA, and the research commenced following ethical clearance from the Department of Industrial and Organisational Psychology at the University of South Africa (ERC Reference 5,668). Participants were initially contacted via email by ECSA on behalf of the researcher and provided with an overview of the study before being invited to take part. Each participant received an informed consent document outlining the terms and conditions of their involvement. Participants were instructed to review and sign the consent form before the scheduled semi-structured interviews. To ensure that participants had sufficient professional experience to provide meaningful accounts, the inclusion criterion required female engineers to have a minimum of 2 years of work experience. A total of nine (9) female engineers who met the inclusion criteria participated in the study (see Table 1).

**Table 1:** Biographical data of the participants.

No	Participant	Race	Age	Qualifications	Work experience
1	A	Black	34	BEng in Civil Engineering	8.5
2	B	Black	31	BEng in Civil Engineering	3
3	C	Black	32	BEng in Civil Engineering	8
4	D	Black	43	Masters Project Management	22
5	E	Indian	32	B. Tech Eng. Structures	8
6	F	Black	35	BSc Civil Engineering	12
7	G	Coloured	52	B Tech	30
8	H	Black	32	BEng Civil Engineering	5
9	I	Indian	32	BSc (Honors) in Electrical Engineering	7

### Data collection

Data were collected through online semi-structured individual interviews by the primary researcher, which lasted between 30 and 45 min each, with nine female engineers at their convenience, using open-ended questions and follow-up probes to explore experiences of remuneration, fairness, career development, mobility, and workplace practices (Creswell and Poth, 2018; DeJonckheere and Vaughn, 2019; Pyo et al., 2023). Interviews were recorded with permission, transcribed verbatim, and conducted privately, with pseudonyms used to protect participants’ identities (Bekwa et al., 2019; Bingham, 2023; Tenny et al., 2020).

Trustworthiness was strengthened through repeated engagement with the data, literature review, triangulation by using other researchers as co-coders, and member checking was done by sharing the themes and subthemes with the participants to support credibility. Rich descriptions enhanced transferability, while reflective notes, self-bracketing, supervisor consultation, and systematic procedures supported confirmability and dependability to minimize researcher bias and subjectivity (Lincoln and Guba, 1985; Nowell et al., 2017; Stahl and King, 2020).

### Procedure

Access to the research site was granted through gatekeeper approval. A gatekeeper letter, signed by the Human Resources Executive, confirming approval to access employee data and to approach engineers for participation, was obtained and submitted with the ethical clearance application. Ethical clearance was obtained from the College of Economics and Management Sciences _ERC Industrial and Organisational Psychology (Ref: 5668), ensuring adherence to confidentiality, voluntary participation, and informed consent procedures.

### Data analysis

The interview data were analysed using inductive thematic analysis within an interpretive phenomenological orientation. The analysis focused on understanding how female engineers experienced and interpreted remuneration fairness and career mobility within their organisational contexts, rather than determining objective equity. Data analysis followed a systematic and iterative process of familiarisation, coding, theme development, review, refinement, and interpretation (Creswell and Poth, 2018). Both manual and computer-assisted methods were used (Bingham, 2023; Cuthbertson et al., 2020), with ATLAS.ti supporting line-by-line coding, memo writing, and visual mapping (Woods et al., 2015; Paulus and Lester, 2021). The researcher independently transcribed and analysed the data (Hammersley and Traianou, 2012), comparing individual accounts to identify shared and divergent patterns. Participant quotations were used to support the themes and demonstrate the link between the empirical data and the researchers’ interpretations.

## Results

The results are displayed using descriptive and themes. The descriptive statistics display the biographical information and frequencies of the participants, while themes show their answers (results) verbatim using subthemes and quotes.

### Descriptive results

Table 1 displays the participants’ biographical information in terms of their pseudonyms, race, age, qualification, and work experience. As per the research objectives and purposive sampling, all the participants were all female.

The sample consisted of 9 female engineers representing civil (majority), structural, and electrical engineering, as well as project management. The participants were predominantly Black (66.7%), had a mean age of 36.8 years, and averaged 11.5 years of professional experience, providing perspectives across different professional and career stages.

### Themes and subthemes

Table 2 displays the themes and subthemes that result from data analysis. There are two themes overall, and both themes have three subthemes.

**Table 2:** Interview question, themes and subthemes.

Interview question	Theme	Subtheme
How do you perceive and experience fairness and equity in remuneration?	Perceived and experienced fairness and equity in remuneration	*Relatively fair* *Procedural inequities* Organisationally mediated negotiation and remuneration fairness
How do the perceived and experienced fairness influence your career decisions, including your motivation to stay in or leave the engineering profession in South Africa?	Role of remuneration fairness and equity in career decisions and retention	*Development-oriented career mobility* Remuneration as a catalyst for turnover intentions among female engineers *Long-term professional goals over equal and fair remuneration*

#### Theme 1: perceived and experienced fairness and equity in remuneration

*Theme 1 emerged from the question*: How do you perceive and experience fairness and equity in remuneration? This question was posed to the female engineers. The subthemes emerged when their answers were coded and grouped.

##### Subtheme 1: relative fairness

The female engineers or participants shared that they perceive their remuneration as fair in comparison with their male peers, implying that they experience equal treatment. The following notable quotes reiterate that:

- “I would say that I am being fairly compensated in comparison to my peers, both male and female. In fact, I would say that I’m towards the upper bracket of the people that I know, people that I studied with, who are at the same level and perhaps the same number of years of working experience.” (Participant F)
- “To be honest, it’s fair but it’s not good, if that makes sense. I’m just in between my peers. I feel like some people would be earning much more than what I am currently earning, so I would think that I’m around the benchmark, but it’s not good. I would say that I don’t think I’m getting what I’m supposed to be getting.” (Participant G)

The female engineers perceived their remuneration as fair or broadly consistent with their expectations and available comparison points of newly appointed females or females in non-STEM sectors. However, these perceptions should not be interpreted as evidence that objective gender-based remuneration disparities were absent within the organization. Rather, the findings indicate that participants’ perceptions of remuneration fairness were shaped by their individual experiences, expectations, and their lack of comparison with male counterparts’ pay. This subtheme provides a divergent case and provide new information that was not expected.

##### Subtheme 2: procedural inequities

The majority of the participants shared that the pay-setting lacks transparency or consistent benchmarking. This led to their negative perceptions of remuneration inequality due to a lack of procedural and informational justice, as noted here:

- “A lot of it is also a human decision because when you start talking about pay range, telling a person that there is a pay range for everyone, but then one or two people are deciding what numbers they put to the range. The manager is the one who’s going to decide when and where am I going to put this person. If it’s dependent on one person and all they have to make sure they do is that they follow within a range.” (Participant D)
- “There should be a thing that’s visible to everyone at this level. This is what you’re getting. It shouldn’t be something that happens behind the scenes it’s the transparency for me.” (Participant H)

The participants are concerned about the lack of transparency on the remuneration model, which leads to negative perceptions.

##### Subtheme 3: organisationally mediated negotiation and remuneration fairness

It appears that for the remuneration to be considered fair, the participants have to negotiate individually, and females tend to accept whatever is offered; hence they experience the remuneration disparities and negative perceptions, as noted here:

- “There was an instance where I sat my manager down and I explained to him ‘OK, this is my life situation, and this is the amount of time I put in’ and then I think that particular year I got a 20% increase.” (Participant E)
- “The answer I got from one of men was that ‘No, we tell them, we speak up’. Whereas women, when we get our increases at the beginning of the year we tend to just say ‘OK, so they only gave me 5%’ but then this one guy was like ‘No I actually go to the director and tell him I’m not happy with the 5% they’re giving everybody’.” (Participant D)

Organisations should ensure that female engineers have equitable access to transparent remuneration processes and meaningful opportunities to raise and review remuneration concerns, thereby reducing reliance on individual negotiation as the primary mechanism for achieving procedural fairness.

#### Theme 2: role of remuneration fairness and equity in career decisions and retention

*Theme 2 emerged from the question*: How do perceived and experienced fairness influence your career decisions, including your motivation to stay in or leave the engineering profession in South Africa? The subthemes emerged when their responses were verbatim transcribed, coded, and grouped.

##### Subtheme 1: development-oriented career mobility

Regardless of the persistent procedural inequities and fair or unfair pay resulting from poor negotiation skills, female engineers in this study remain employed, and the only time they resign or leave is to advance their careers, gain more experience in a specific niche, and become flexible.

- “If I’m being honest, I just resigned for another opportunity because as much as the pay is fair, I think you’ll have to think about your overall potential. I want to make sure that I am skilled in at least one area of my work, so that there is longevity to my career, you know? I’m moving now to get experience in the app sector. Which I feel was lacking in my CV just so that I remain relevant.” (Participant A)
- “I think experience would be the big consideration in me leaving and then maybe more diverse or technical projects rather than the ones that we do which are quite simplistic. I want a bit more experience in a more technical industry or something that is more innovative, if I can call it that. It wasn’t necessarily my passion or what I wanted to do; it was a niche that I filled.” (Participant C)
- “I think the factors that has pushed most women out hasn’t been money, I think it’s been access to work or projects, personality clashes with bosses and flexibility.” (Participant F)

These notions imply that female engineers are content with either fair or unfair pay within the engineering company. This makes development-oriented career mobility a valid reason for female engineers in this study to resign, along with other popular reasons. Perceptions and experiences of fairness, negative or positive, in remuneration do not influence their career movement. These findings are new in the STEM field.

##### Subtheme 2: remuneration as a catalyst for turnover intentions among female engineers

While remuneration is not always the primary driver of turnover intentions, inadequate or inequitable pay increases employees’ openness to external opportunities. The findings also suggest that salary operates alongside other workplace factors such as career development, access to meaningful work, treatment by management, and workplace flexibility.

- “Well, it does play on your psyche because you start looking outside. You start being curious to know what else is out there. I was actually thinking about it and mentioned to a friend, when you’re feel like you’re compensated fairly, even when you get those head-hunting inboxes on LinkedIn, you don’t look at them, you say ‘I’m fine, thank you’ but then even if you love the company you will at least ask ‘How much are you offering’. So there’s a little bit of that curiosity to say, OK, but you know, especially if it’s a company that you enjoy and you wouldn’t want to leave given the option if like if the work environment is fine and you feel like you’re growing, you feel like there’s a lot more opportunities to grow, the lack of remuneration inspires you to check outside what’s available because you also don’t want to pass up an opportunity because you like the culture.” (Participant D)
- “I think to be honest, the first thing that I think about even as a as an individual is that if I get a better offer, I’ll leave. So, I think if somebody offers me better than what my current employer is currently giving to me, I think I would leave.” (Participant F)
- “I don’t know, maybe I’m a bit different, but money hasn’t been the primary reason of my consideration to stay or not stay. In the eight years I’ve been at the company, I have thought about the financial aspect of things but the thing that’s kept me is the access to projects, or career progression, whatever you want to call it. I will say if today a company came and told me they were going to pay double, I would be out today. If what is being on offer outside of begging far exceeds what I’m getting now, it is a great motivator for me to leave.” (Participant F)
- “Sometimes I think of switching from engineering something else maybe mining engineering is better, and I know someone with the same years of experience, same qualifications that works in mining and this person earns much more than I do, there’s a huge difference.” (Participant H)
- “I don’t think the salary difference will make me leave the company. I think it’s something I can always negotiate with the company the only reason I would leave the company if I were not happy, or not being treated well, not because of the salary—that I can always negotiate.” (Participant B)
- “I think the factors that has pushed most women out hasn’t been money, I think it’s been access to work or projects, personality clashes with bosses and flexibility.” (Participant F)

The findings reveal that salary, rather than being a direct cause of resignation, inadequate remuneration often acts as a trigger that encourages female engineers to evaluate external opportunities.

##### Subtheme 3: Long-term professional goals over equal and fair remuneration

The data indicate that female engineers often adopt a calculated approach to career mobility, where remuneration is considered alongside broader career objectives. Decisions to leave are frequently delayed until participants perceive that they have acquired the necessary skills, experience, or professional exposure to support their next career move, as noted here:

- “When I look at my career, it’s not solely dependent on the remuneration itself, it’s also based on other factors like the growth that I get from this place and is linked to the culture in the company, it’s linked to the responsibilities or the exposure that I still need, it’s based on that. I wouldn’t limit it to just the remuneration because my goal is to get the experience that I need, eat from this place and as much as I can, and then look at the next step to my career.” (Participant I)
- “There’s a lot that comes into making the decision of leaving because you ask yourself ‘where you are going, are things not the same as here?’. You ask yourself all that and now you start to think to yourself, ‘what other motivation do I have besides the salary?’ And for me personally, the other motivations, are my career—until I feel satisfied that I have learned everything I can learn from this place or I have all the exposure that I need, then I can decide what’s next for my career but in terms of the remuneration for me personally I would say that is not the number one factor.” (Participant I)
- “I’m working in a project management role, I will do designs as they come up in my division, when it is required, but because there are so many projects, it’s a full-time job just managing and sorting out the invoices and making sure everyone performs, and the reports are reviewed and writing and collecting all the information. It’s a three-year project but if it were to extend another year over that, I wouldn’t want to necessarily be in a project management role. I would prefer to go back to design because I filled in the gap when they needed it and then they saw it as needing me, which is why every time I was unhappy, they gave me a raise, but I don’t think that that’s necessarily where I see myself as a project manager.” (Participant C)
- “I think I am happy at the company because they have indicated that once I do get my PR, they’re going to make the salary adjustment, but it’s still in the future, you know what I mean? It’s a promise for something to come. If they don’t make good, if I can call it that, on that promise then I would obviously consider leaving.” (Participant C)
- “I’ll give an example, someone is has recently resigned, a woman and she’s going to be working from home so that’s a big factor. Was she fairly compensated, I can’t say but I can tell you that if even if she was being unfairly compensated, the likelihood is that she wouldn’t have left because of that, the draw is more other things rather than it’s than it is money?” (Participant F)

Female engineers are less concerned about equal remuneration but more concerned about their career within the organisations and commitment to future rewards and recognition. If these are not promised, they strategically plan to leave.

## Discussion

This study aimed to explore the female engineers’ perceptions and experiences of remuneration fairness and inequity and how remuneration influences their retention and career progression. The findings discussed in terms of the themes and subthemes are as follows.

The findings reveal that female engineers at the surveyed firm perceive and experience their compensation as fair or broadly consistent with their expectations. These findings are unexpected but, according to Jiang (2021), this phenomenon arises from the fact that entry- and mid-level jobs in STEM pay highly, leading to females being very satisfied with their pay, regardless of the possible pay differences with male counterparts. This relates well to the SET and perceived or experienced organizational justice, whereby pay-satisfied females become loyal to the organisations, leading to low turnover and career satisfaction (Chen et al., 2023; Mabaso, 2026).

Furthermore, the findings revealed that, although not being specific about gender pay disparities, females experience pay secrecy or a lack of remuneration transparency that leads them to perceive the whole process as unfair (Cruz et al., 2026; Zajac et al., 2024). Conclusively, these findings imply negative perceptions and experiences and may lead to high turnover and disrupted career moves (Hasyim and Bakri, 2024; Mabaso and Mdluli, 2025; Stofberg et al., 2022). According to the OJT, when employees perceive secrecy in how remuneration decisions are made, they are likely to perceive and experience unfairness and inequity (Colquitt et al., 2001; Cruz et al., 2026; Greenberg, 1987; Zajac et al., 2024).

Moreover, the findings indicated a need for organisationally mediated negotiation and remuneration fairness to achieve procedural justice towards equal remuneration. The findings extend the understanding of remuneration fairness by demonstrating that negotiation should be situated within the organisational structures and gender relations in which it occurs (Colquitt, 2001; Mabaso, 2026). Rather than conceptualising negotiation as an individual competency that employees either possess or lack, the findings indicate that negotiation is socially and organisationally embedded (Bowles et al., 2007; Mazei et al., 2015). According to Amanatullah and Morris (2010) and Eagly and Wood (2012), in male-dominated engineering environments, hierarchical relationships, gendered expectations and unequal access to organisational decision-makers may influence both the willingness to initiate remuneration discussions and the outcomes of those discussions. Consequently, remuneration disparities cannot be adequately explained by differences in individual negotiation behaviour alone. A more comprehensive interpretation recognises the interaction between employee agency and organisational conditions, whereby employees may exercise agency while organisational structures simultaneously enable or constrain the effectiveness of that agency, as concluded by Ridgeway (2011).

Findings from the second part of the objective, which was to further explore the role of remuneration fairness in career decisions and retention, reveal that female engineers would not really leave an organisation due to dissatisfaction with pay, but they can leave due to unsatisfactory career progress or advancement. Fouad et al. (2017) concluded that women in the engineering field value recognition at work and adequate opportunities for advancement more than fair remuneration. This makes development-oriented career mobility a valid reason for female engineers in this study to resign, along with other popular reasons. Conclusively, it appears that female engineers in this study choose to pursue career moves to access better professional growth, learning opportunities, and career advancement, rather than leaving solely because of remuneration dissatisfaction (O’Neil et al., 2013). Perceptions and experiences of fairness, negative or positive, in remuneration do not influence their career movement. These findings are new in the STEM field. Furthermore, the female engineers appear not to be concerned about remuneration gaps turnover because of the secrecy or lack of transparency, but when actually experienced, the pay disparities lead to turnover intentions because it becomes a trigger that encourages female engineers to evaluate external opportunities, as supported by Abdalnour et al. (2023) and Fouad et al. (2017). Furthermore, the results show that even when organisational culture, growth opportunities, and job satisfaction are positive, experienced dissatisfaction with remuneration encourages employees to explore the labour market. This suggests that compensation serves as an important retention factor (Mabaso and Mdluli, 2025). Notably, remuneration alone may not determine retention, but substantial pay disparities can outweigh other positive organisational attributes (Mabaso, 2026). The findings further introduce comparisons with peers in line with SET, OTJ, and equity theories, where employees evaluate their compensation relative to others for fairness, justice, and equity and adjust their attitudes and behaviours accordingly (Chen et al., 2023; Mabaso, 2026). However, when poor working conditions are rife, remuneration may function as a “pull factor” toward external opportunities, while workplace experiences serve as “push factors” that drive employees away from the organisation (Çakır et al., 2026; Yao et al., 2026). In contrast, some females do not perceive salary differences as a sufficient reason to leave, and placing greater emphasis on management’s treatment and overall job satisfaction. This indicates that not all female engineers prioritise compensation equally, and retention decisions are influenced by a broader set of organisational experiences supported by Mabaso (2026).

Persistent pay disparities lead to strategic career decisions being made by females, whereby they intentionally stay with the company while learning and absorbing as much information as possible before making a big career move. Collectively, the findings suggest that female engineers do not make impulsive decisions based solely on remuneration disparities, in line with findings by Dos Santos (2022) and McGregor et al. (2017). Instead, they engage in strategic career planning, carefully timing transitions to coincide with the achievement of developmental goals, the completion of key projects, the attainment of professional credentials, or the exhaustion of learning opportunities within their current organization (Ketchiwou et al., 2022; Ketchiwou and Van der Walt, 2023). Remuneration equity and fairness remain important considerations, particularly when linked to recognition for achievements and future advancement, but they are evaluated within a broader framework of career progression, role alignment, organisational trust, and flexibility. The findings therefore indicate that turnover intentions among female engineers are often delayed or moderated when organisations continue to provide valuable developmental opportunities and demonstrate a commitment to future reward equity (O’Neil et al., 2013). However, when promises of fair remuneration are not fulfilled, career aspirations become misaligned with organisational roles, or alternative opportunities offer greater flexibility and growth, female engineers become more likely to initiate a strategically timed career transition. This highlights that retention is not solely dependent on competitive pay but on organisations’ ability to support employees’ long-term career objectives while maintaining perceptions of fairness and trust (Chen et al., 2023; Çakır et al., 2026; Mabaso, 2026; Yao et al., 2026).

### Limitations

The study had three main limitations. First, the limited number of female engineers in the organisation restricted the sampling frame and the diversity of experiences captured. Second, the absence of male engineers prevented direct gender comparison and limited the extent to which reported remuneration experiences could be attributed specifically to gender rather than other factors such as tenure, experience, occupational level or performance. Third, limited information about the organisation’s remuneration structures, salary-setting procedures and broader gender context constrained the triangulation of participants’ perceptions with organisational evidence. The findings should therefore be interpreted as reflecting participants’ experiences and perceptions within the specific organisational context rather than as evidence of generalisable gender-based remuneration inequality. As with any qualitative research, the researcher’s subjectivity plays a role in the coding and interpretation of data (Creswell and Poth, 2018). Despite efforts to ensure rigour through reflexive journaling and transparency in the analytical process, interpretations are shaped by the researcher’s positionality and lens (Stahl and King, 2020).

### Implications

This study offers valuable insights, with direct implications for organisational practice, HR management, and equity-driven policy development. By exploring female engineers’ perceptions of fairness and equity in remuneration, the research highlights key areas where organisational systems and cultures may unintentionally perpetuate gender-based disparities. One key implication is the need for organisations, particularly in STEM industries, to enhance transparency in remuneration processes and clearly communicate how pay decisions are made. The findings suggest that a lack of clarity often fuels perceptions of unfairness and undermines trust in leadership.

The study underscores the importance of empowering employees with negotiation agency through training, mentorship, and inclusive leadership practices. Organisations may need to re-evaluate existing performance and reward systems to ensure they do not inadvertently disadvantage female professionals or reinforce traditional gender roles. The insights from this research can also inform human resource policies, especially those relating to pay equity audits, promotion criteria, and grievance procedures. Incorporating employee perceptions into such processes can help create more equitable and responsive organisational cultures. Furthermore, the findings can guide leadership development programs to raise awareness of unconscious bias in decision-making and foster more inclusive management practices. This is particularly relevant in male-dominated environments where diversity and equity initiatives often face resistance or tokenism. The study provides a foundation for actionable interventions aimed at improving retention, satisfaction, and career advancement opportunities for women in engineering, contributing to broader goals of gender equity in the workplace and the sustainable development of talent in technical professions.

### Conclusions and recommendations

The contribution of this study lies not in determining the existence or absence of an objective gender pay gap, but in examining how female engineers experience and interpret remuneration fairness within their organisational context. The findings indicate that perceptions of fairness are shaped by individual expectations, comparison points, career aspirations, workload, access to development opportunities, and interactions with management. Notably, participants appeared to prioritise career advancement, experience, and professional growth, sometimes over concerns about remuneration fairness or working conditions. This may result in limited salary negotiation or assumptions that existing remuneration is competitive, potentially reducing awareness of disparities and delaying recognition of inequitable practices.

These findings highlight the importance of organisational interventions that support both career advancement and remuneration equity. Employers should strengthen career development opportunities, recognise employees’ contributions through transparent promotion and reward systems, and encourage open discussions about remuneration. Greater pay transparency and awareness of market-related remuneration may also enable female engineers to make more informed decisions regarding their careers and compensation by making public the salary ranges or notches. In addition, organizational policies should make it possible for individuals to negotiate for their own salaries. Moreover, gender-equity awareness programmes among management and males could contribute to a more transparent and inclusive organisational culture. Overall, addressing both the career aspirations and remuneration experiences of female engineers may enhance job satisfaction, retention, and gender equity within engineering and the broader STEM environment.

## Data Availability

The raw data supporting the conclusions of this article will be made available by the authors, without undue reservation.
